# Assessment of Isometric Shoulder Strength in Swimmers: A Validation and Reliability Study of the ASH and iASH Tests

**DOI:** 10.3390/jfmk10010092

**Published:** 2025-03-12

**Authors:** Hugo Ogando-Berea, Santiago Virgós-Abelleira, Pablo Hernandez-Lucas, Fernando Zarzosa-Alonso

**Affiliations:** 1 LabEndo Research Group, Department of Functional Biology and Health Sciences, University of Vigo, Campus Lagoas-Marcosende, 36310 Vigo, Spain; hoberea@uvigo.gal; 2 Faculty of Education and Sport Science, University of Vigo, 36005 Pontevedra, Spain; yago.virgos@gmail.com (S.V.-A.); fzarzosa@uvigo.es (F.Z.-A.); 3 HI10 Research Group, Department of Functional Biology and Health Sciences, Faculty of Physiotherapy, University of Vigo, Campus A Xunqueira, 36005 Pontevedra, Spain

**Keywords:** measurement, movement analysis, functional assessment, sport, exercise

## Abstract

**Background/Objectives**: Shoulder pain is one of the most common injuries among athletes who perform overhead movements. The Athletic Shoulder Test (ASH) has been validated to measure isometric shoulder strength in rugby and baseball players but has not yet been applied to swimmers, where the prevalence of shoulder pathologies reaches up to 91%. The present study aims to validate the ASH and Inverse Athletic Shoulder Test (iASH) in swimmers and establish general values for both tests. **Methods**: A total of 21 swimmers from the Galician and Asturian Swimming Federation were evaluated using the ASH and iASH tests, measuring strength in three positions (180°, 135°, and 90°). Relative reliability was analysed using the Intraclass Correlation Coefficient (ICC), absolute reliability was assessed through the Standard Error of Measurement (SEM) and Minimal Detectable Change at 90% confidence (MDC90), and variability was determined using the Coefficient of Variation (CV%), applying statistical tests such as Wilcoxon. **Results**: The ASH and iASH tests demonstrated excellent reliability (ICC = 0.9) across all positions, with acceptable variability (CV% < 35%). No statistically significant differences were found between the preferred and non-preferred side (*p* > 0.05), except in the iASH test at 180°, where a difference was observed (*p* = 0.007). The SEM values ranged from 4.39 to 7.39 N, while the MDC90 varied between 10.22 and 17.19 N, ensuring the tests’ sensitivity in detecting real changes in shoulder strength. **Conclusions**: The ASH and iASH tests are reliable tools for assessing shoulder strength in swimmers and can be used to monitor muscular imbalances and prevent injuries. The symmetry in strength between both sides supports their applicability in preventive programmes.

## 1. Introduction

Shoulder pain is one of the most common injuries among athletes who perform overhead movements [1]. To minimise this issue, assessments have been proposed that comprehensively address the functional localisation of the technical gesture. In this regard, Ashworth, B. et al. [2] introduced in 2020 the test known as the “Athletic Shoulder” test (ASH), designed to measure isometric strength in shoulder extension at various angles (180°, 135°, and 90°) in rugby players [2], and its application has subsequently been explored in other sports, such as baseball [3]. However, to date, this assessment tool has not been applied to swimmers, despite the prevalence of shoulder pathologies in this population reaching up to 91% [4,5,6,7] and ranging between 41% and 51% in young swimmers [8,9].

During the stroke, swimmers’ shoulders are subjected to repetitive movements of internal rotation, abduction, and extension, leading to increased strength in these muscle groups compared to the general population [10,11,12,13]. These selective increases may generate muscle imbalances over time [14]. In this context, the relationship between shoulder pain and strength ratios measured via dynamometry in internal and external rotations has been studied, identifying this imbalance as one of the predisposing risk factors for the development of “Swimmer’s Shoulder”, along with fatigue [15,16], overall shoulder weakness, training load, and injury history [9,17,18,19]. Although normative data exist for swimmers regarding strength ratios in internal and external rotation, as well as for arm flexion and extension [11], there is still no information regarding different locations throughout the stroke cycle an aspect of particular interest in preventing potential imbalances during the execution of the technical movement. In this context, we consider that the ASH test could be highly useful, given that the phase between 180° and 90°, corresponding to the catch phase, is where the greatest force is exerted and, consequently, the risk of impingement and shoulder injury increases [20]. Moreover, determining the agonist-antagonist strength ratios in each phase of the stroke cycle is relevant, as it would enable the optimisation of prevention programmes for swimmers [21].

For these reasons, the objectives of the present study are: (1) The validation of ASH and iASH tests in swimmers and (2) the establishment of general values for both tests, with the hypothesis that the ASH and iASH tests will be valid and reliable methods for assessing isometric shoulder strength in swimmers, providing general reference values that will enable the detection of muscular imbalances and the optimization of injury prevention programmes.

## 2. Materials and Methods

### 2.1. Design

This cross-sectional validation and reliability study involved 21 swimmers from the development programme of the Galician and Asturian Swimming Federation, who participated in the ASH test and the newly proposed inverse ASH (iASH) test. Prior to inclusion, all athletes signed an informed consent form; for minors, consent was provided by their legal guardians, ensuring compliance with ethical regulations and the principles established in the Declaration of Helsinki. The experimental procedures adhered to the CONSORT guidelines. The study protocol was registered on ClinicalTrials.org (code: NCT06763107) and received approval from the Ethics Committee of the Faculty of Sport and Physical Activity Sciences in the meeting held on 22 March 2023, being assigned the code: 05-220323.

The person responsible for analysing the statistical data was blinded to the group assignments to minimise potential bias and ensure the objectivity of the results.

### 2.2. Inclusion and Exclusion Criteria

The inclusion criteria were swimmers with more than 15 h of training per week, aged between 14 and 18 years, with an absence of acute injuries in the cervical or scapular region, and the ability to perform the ASH and iASH tests without compensatory movements. The exclusion criteria included: the presence of injuries in the cervical or scapular region (such as glenohumeral instability, rotator cuff tendinopathy, subacromial bursitis, subacromial impingement, SLAP lesion, dislocations, sprains, etc.) and/or the inability to maintain an appropriate position during the tests due to insufficient mobility or strength, and refusal to sign the informed consent (or that of the legal guardian in the case of minors).

### 2.3. ASH and iASH Tests

The tests were conducted in a controlled environment within the sports facilities of the Galician Swimming Federation, ensuring a stable ambient temperature of 22 ± 1 °C, uniform lighting, and low noise conditions to minimise distractions. All measurements were performed at the same time of day (16:00–18:00) to reduce potential circadian rhythm effects on performance. The ASH and iASH tests were conducted by a graduate in physical activity and sport, specialising in strength and biomechanics assessment.

For the execution of the ASH and iASH tests, three lines were marked on the floor with adhesive tape to indicate the abduction angles of 180°, 135°, and 90°. Participants were positioned on six stacked mats to align their height with the Chronojump force sensor (Chronojump, Boscosystem^®^, Barcelona, Spain).

**Figure 1:**
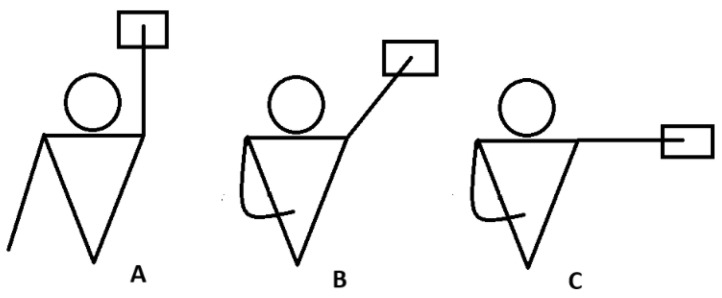
ASH test. (**A**) (180°); (**B**) (135°); (**C**) (90°).

During the ASH test, swimmers were placed in a prone position and performed shoulder extensions in three positions: I (180°), Y (135°), and T (90°). Each athlete performed three 3-s trials in each position and with each arm, resting for 20 s between trials and alternating arms. Throughout all tests, the elbows remained fully extended, using the heel of the hand as the contact point with the sensor [2] (Figure 1).

**Figure 2:**
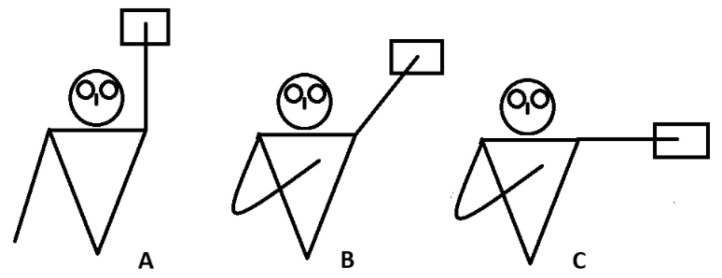
iASH test. (**A**) (180°); (**B**) (135°); (**C**) (90°).

In the iASH test, participants were positioned in a supine position and performed shoulder flexions in the same three positions. The procedure was similar but used the back of the hand as the contact point with the sensor. For both tests, standardised conditions were ensured, and athletes were instructed to avoid compensatory movements that would invalidate the attempt (Figure 2).

### 2.4. Study Timeline

The study was conducted over a period of 18 months. During the first year, a pilot analysis was carried out with a small sample of 10 participants, allowing for adjustments and optimisation of the research protocol. Subsequently, the protocol was registered and approved, after which swimmer recruitment took place. Initial assessments were conducted in December 2024, and the ASH and iASH tests were performed on two consecutive days under identical testing conditions. Finally, the interpretation of the results and the manuscript preparation were completed in February 2025.

### 2.5. Statistical Analysis

Descriptive statistics were calculated for the Peak Force (NPF) in each position and for both evaluation days, expressed in Newtons (N) as mean and Standard Deviation (SD). Relative reliability was assessed using Intraclass Correlation Coefficients (ICC), while absolute reliability was evaluated through the Standard Error of Measurement (SEM), the Minimum Detectable Change at 90% confidence (MDC90), and the Typical Error (TE). The Coefficient of Variation (CV%) was used to analyse the relative dispersion of the data.

The consistency of measurements between days was assessed using the Wilcoxon test for related samples, considering a significance level of *α* = 0.05. Additionally, differences in NPF measurements between the preferred and non-preferred sides were evaluated using the Wilcoxon test again. Finally, ASH/iASH ratios were calculated as indicators of strength relationships between both tests. All statistical analyses were performed using R software (version 4.2.1, R Core Team, 2021).

## 3. Results

The study included 21 swimmers, comprising 11 males and 10 females. The mean age was 16.76 ± 1.09 years, with a distribution of 16.73 ± 1.19 years for males and 16.80 ± 1.03 years for females. The overall mean height was 176.57 ± 8.44 cm, with males measuring 181.64 ± 6.53 cm and females 171.00 ± 6.70 cm. The mean body weight was 66.43 ± 7.63 kg, with an average of 70.91 ± 4.78 kg in males and 61.50 ± 7.25 kg in females. Regarding the preferred breathing side, 20 participants favoured the right side, while one participant preferred the left side (Table 1).

The results of the ASH test are detailed in Table 2. Three angular positions were evaluated: I (180°), T (135°), and Y (90°). In the ASH test, no statistically significant differences were observed between measurements taken on Day 1 and Day 2 for any of the evaluated positions. In position I, W = 107.0, *p* = 0.785; in position Y, W = 106.0, *p* = 0.759; and in position T, W = 93.0, *p* = 0.452. Additionally, for each position, the means and Standard Deviations (SD) of Peak zforce (NPF) in Newtons (N) were recorded over two evaluation days.

For all evaluated positions in the ASH test, the Coefficient of Variation (CV%) remained below 35%, indicating acceptable variability in the measurements. The Typical Error (TE) ranged from 0.04 to 2.11, reflecting the precision of individual measurements. The Intraclass Correlation Coefficient (ICC) of 0.9 across all positions suggests excellent test reliability.

The Standard Error of Measurement (SEM) ranged from 5.34 to 7.39 N, indicating adequate precision in muscle strength measurement. The Minimum Detectable Change at 90% confidence (MDC90), which ranged from 12.43 to 17.19 N, represents the smallest variation in strength that can be considered real rather than attributable to measurement error. Finally, the MDC%, which ranged from 20.83% to 25.3%, reflects the magnitude of detectable change relative to the mean measurements (Table 2).

The results of the inverse ASH test are presented in Table 3. As in the ASH test, three angular positions were evaluated. In the inverse ASH test, no significant differences between days were identified. For position I, W = 84.0, *p* = 0.288; for position Y, W = 115.0, *p* = 1.000; and for position T, W = 77.0, *p* = 0.191. Additionally, the CV% ranged between 28.32% and 34.84%, indicating acceptable variability similar to that observed in the ASH test. The TE ranged from 0.1 to 1.48, reflecting slightly higher measurement precision compared to the ASH test. The ICC of 0.9 across all positions confirms the excellent reliability of the inverse ASH test. The SEM in the inverse ASH test ranged from 4.39 to 5.16 N, demonstrating adequate precision similar to that of the ASH test. The MDC90 varied between 10.22 and 12.0 N, while the MDC% ranged between 20.83% and 25.63%, indicating that the detectable changes are consistent and comparable to those of the ASH test.

Table 4 presents the differences between the preferred and non-preferred side for each position and test type, differentiated globally and by gender. In the analysis of differences, conducted using the Wilcoxon test, the following results were observed:

In the ASH test, no statistically significant differences were found between both sides in any of the evaluated positions. For position I, the Wilcoxon statistic was W = 101, with a *p*-value of 0.633. Among males, it was W = 55 and *p* = 0.76, while among females, it was W = 45 and *p* = 0.818. In position Y, the Wilcoxon statistic was W = 115, with a *p*-value of 1. Among males, W = 66 and *p* = 1, and among females, W = 36 and *p* = 0.625. In position T, the statistic was W = 109 with a *p*-value of 0.838. Among males, W = 57 and *p* = 0.895, and among females, W = 41 and *p* = 0.625.

In the iASH test, significant differences were identified between the preferred and non-preferred side only in position I, where the Wilcoxon statistic was W = 40, with a *p*-value of 0.007. Among males, W = 21 and *p* = 0.054, while among females, W = 15 and *p* = 0.193. In position Y, no significant differences were found, with a Wilcoxon statistic of W = 109 and *p* = 0.838. Among males, W = 55 and *p* = 0.705, and among females, W = 35 and *p* = 0.953. For position T, the statistic was W = 98, with a *p*-value of 0.562. Among males, W = 50 and *p* = 1, and among females, W = 38 and *p* = 0.625.

The strength ratios between the ASH and iASH tests are presented in Table 5. These ratios were calculated by dividing the mean ASH test value by the mean iASH test value for each evaluated position.

In position I, the global mean ratios were 1.73 (SD = 0.42) for the preferred side and 1.79 (SD = 0.41) for the non-preferred side, with a *p*-value of 0.23 (W = 80). Among males, the mean ratios were 1.66 (SD = 0.32) and 1.76 (SD = 0.42), respectively, with a *p*-value of 0.18 (W = 17). Among females, the mean ratios were 1.81 (SD = 0.51) and 1.83 (SD = 0.41), with a *p*-value of 0.77 (W = 24).

In position Y, the global mean ratios were 1.40 (SD = 0.24) for the preferred side and 1.41 (SD = 0.27) for the non-preferred side, with a *p*-value of 1.00 (W = 115). Among males, the values were 1.42 (SD = 0.25) and 1.37 (SD = 0.32), with a *p*-value of 0.83 (W = 30). Among females, the mean ratios were 1.38 (SD = 0.24) and 1.45 (SD = 0.22), with a *p*-value of 0.56 (W = 21).

In position T, the global mean ratios were 1.20 (SD = 0.24) for the preferred side and 1.21 (SD = 0.20) for the non-preferred side, with a *p*-value of 0.32 (W = 86). Among males, the values were 1.18 (SD = 0.23) and 1.20 (SD = 0.23), with a *p*-value of 0.24 (W = 19). Among females, the mean ratios were 1.23 (SD = 0.26) and 1.23 (SD = 0.17), with a *p*-value of 0.77 (W = 24).

## 4. Discussion

The objectives of the present study were to validate the ASH test in swimmers, propose and validate the inverse iASH test, and establish reference values for both tests.

Among the main findings, it stands out that relative reliability, assessed through the ICC, reached a consistent value across all tests and positions, indicating excellent reliability [22,23] and demonstrating the high consistency of measurements across different evaluation days [22]. On the other hand, absolute reliability, evaluated through SEM and MDC90, showed low and consistent values [24,25], supporting the precision and the ability to detect real changes in shoulder muscle strength. The CV% remained within a range considered acceptable in all tests, indicating controlled variability and allowing reliable comparisons between different datasets [26]. Additionally, the TE demonstrated adequate precision in individual measurements, consistently remaining low in both tests, confirming that the measurements closely approximate the true value. Furthermore, the MDC90 helped establish the minimum threshold for significant change, ensuring that observed variations reflect true changes in muscle strength rather than simple fluctuations due to measurement error [25].

These findings support the convergent validity of the ASH and iASH tests, as the strength relationships between both tests are consistent and predictable, allowing them to be used complementarily to assess strength balance in swimmers’ shoulders. Notably, the obtained values are comparable to those reported by Ashworth et al. [2] in terms of ICC, SEM, MDC90, TE, and CIM for both the ASH and iASH tests.

Regarding the absolute strength values obtained during the ASH test, our study’s results were considerably lower than those initially reported in rugby players [2] and slightly lower than those found in baseball players [3]. This difference may be attributed to several factors: the younger age of our sample, differences in height and body mass, the inclusion of female participants in our study, and the specific demands of the sport. When comparing the extension and flexion data in position I (180°) of young swimmers with similar characteristics to our sample, the results obtained for both the ASH and iASH tests were more comparable, albeit slightly lower.

It is important to note that in the study by McLaine et al. [11], flexion was measured at an angle slightly lower than 180° (approximately 165°) and using manual resistance dynamometry, which may introduce an additional error factor due to the evaluator’s applied resistance. In contrast, our procedure involved measurement against a fixed point (the floor), which could explain the small discrepancy in the obtained values.

To date, the literature has not reported isometric strength ratios for this population group in different stroke positions beyond those obtained in studies focused on rotation and flexion-extension in the catch position, such as the study by McLaine et al. [11]. In that study, the strength ratios between internal and external rotators, as well as between extensors and flexors, were lower compared to those obtained in our analysis. In particular, the strength ratio in position I stood out as the highest in our study. In positions Y and T, the values found were more similar to those previously reported, although still slightly higher [11]. In another study by Drigny et al. [27], internal and external rotation strength was measured concentrically and eccentrically. The results showed a similar trend to the values obtained in our study, although these data correspond to dynamic strength rather than isometric strength. However, they differed from those reported by McLaine et al. for the same rotational movement [11].

Regarding statistical comparison, the analysis between the preferred and non-preferred side in both the ASH and iASH tests, as well as between different measurement days, did not reveal significant differences except for a single value. This significant difference was observed in the iASH test between the breathing side and the opposite side, specifically in position I (180°). This finding could be explained by the fact that this angle represents the moment of maximum support before initiating propulsion [28,29]. It is the only mechanically distinct point in the stroke when comparing the breathing side with the opposite side, as the stroke pattern changes when bearing more weight while lifting the head to breathe [28].

It is recognised that once the Swimmer’s Shoulder syndrome is established, permanent changes occur in the activation pattern of the muscles involved in swimming, altering the comparative biomechanics between swimmers with and without the syndrome [20]. Over time, this may lead to a high prevalence of structural changes in the rotator cuff and biceps tendons, which has been linked to an increased occurrence of symptoms [30,31]. Additionally, tendinosis is observed more frequently in swimmers with a positive sulcus sign, suggesting a role for shoulder laxity [31]. Therefore, having reference values for an injury-free sample of swimmers is particularly important [32].

Several limitations should be considered when interpreting the results of this study. Firstly, the small sample size limits the generalisability of the findings, as does the relative homogeneity in age and sex of the participants, since these factors significantly affect musculoskeletal properties. The inclusion of adolescents aged 14–18, both males and females, restricts the extrapolation of these results to adult or professional swimmers. Additionally, the study did not account for the hormonal variations inherent to adolescent development, nor did it specifically control for the menstrual cycle phase in female participants, factors which could significantly influence muscular strength performance. To partially mitigate these limitations, the study adopted rigorous standardised assessment protocols, including consistent timing, adequate rest intervals, repeated assessments on consecutive days, and clearly defined inclusion criteria.

Future lines of research should focus on substantially expanding and diversifying the sample, incorporating swimmers from different age groups, sexes, and competitive levels. It would also be beneficial to conduct targeted studies assessing the impact of hormonal variations, particularly menstrual cycle phases in female athletes, on shoulder strength measurements. Furthermore, longitudinal research is recommended to prospectively investigate the predictive capability of ASH and iASH tests in relation to shoulder injury occurrence, thereby enhancing the practical effectiveness of strength-based preventive interventions in swimmers.

This study provides significant clinical implications. Firstly, the validation of the ASH test and the newly developed iASH test offer useful and reliable tools to assess shoulder muscle strength in swimmers. Furthermore, establishing specific reference values for this population allows for the early detection of muscular imbalances, which is crucial for preventing common injuries such as Swimmer’s Shoulder. Additionally, detailed assessments at different angular positions enable therapists and coaches to design targeted preventive and strengthening programmes. Routine implementation of these tests could optimise athletic performance and significantly reduce the incidence of shoulder injuries among swimmers. Nevertheless, the authors again acknowledge that despite these promising findings, the small sample size represents an important limitation, and further studies are necessary to confirm and expand upon these initial results.

## 5. Conclusions

The results obtained demonstrate that both the ASH and iASH tests are reliable and consistent methods for assessing shoulder muscle strength in swimmers. The high reliability and acceptable variability in both tests suggest that these methods can be effectively used to monitor shoulder strength balance, which is crucial for preventing shoulder pain-related injuries in swimmers. Additionally, the absence of significant differences between the preferred and non-preferred breathing side supports the muscular symmetry of the evaluated swimmers’ shoulders.

Overall, these findings support the complementary use of the ASH and iASH tests to obtain a comprehensive assessment of shoulder muscle strength balance, facilitating the implementation of targeted preventive strategies for muscle groups identified as being at higher risk for shoulder pain development.

## Figures and Tables

**Table 1:** Participant characteristics.

Participant	Weight (kg)	Height (cm)	BMI	Age (Years)	Side of Breathing
Overall (*n* = 21)	66.43 ± 7.63	176.57 ± 8.44	21.23 ± 1.17	16.76 ± 1.09	20 Right—1 Left
Males (*n* = 11)	70.91 ± 4.78	181.64 ± 6.53	21.49 ± 0.97	16.73 ± 1.19	11 Right
Females (*n* = 10)	61.50 ± 7.25	171.00 ± 6.70	20.97 ± 1.36	16.80 ± 1.03	9 Right—1 Left

BMI: Body Mass Index.

**Table 2:** ASH test results.

Preferred Breathing Side	Position	Mean Day 1	SD Day 1	Mean Day 2	SD Day 2	CV (%)	TE	CIM	ICC	SEM	MDC90	MDC%
Not	I (180°)	77.86	23.36	77.9	25.08	30.01	0.04	0.02	0.9	7.39	17.19	22.08
	Y (135°)	64.39	22.14	62.27	18.75	34.39	2.11	1.06	0.9	7.0	16.29	25.3
	T (90°)	57.51	16.9	58.23	17.01	29.38	0.72	0.36	0.9	5.34	12.43	21.62
Yes	I (180°)	78.52	21.66	80.37	26.7	27.58	1.86	0.93	0.9	6.85	15.93	20.29
	Y (135°)	64.48	19.4	66.07	21.62	30.09	1.59	0.79	0.9	6.14	14.28	22.14
	T (90°)	57.47	14.21	58.5	16.07	24.73	1.03	0.52	0.9	4.49	10.45	18.19

SD: Standard Deviation; CV (%): Coefficient of Variation; TE: Typical Error; CIM: Confidence Interval of the Mean; ICC: Intraclass Correlation Coefficient; SEM: Standard Error of Measurement; MDC90: Minimal Detectable Change at 90% Confidence; MDC%: Minimal Detectable Change Percentage.

**Table 3:** iASH test results.

Preferred Breathing Side	Position	Mean Day 1	SD Day 1	Mean Day 2	SD Day 2	CV (%)	TE	CIM	ICC	SEM	MDC90	MDC%
Not	I (180°)	44.79	14.13	46.05	14.06	31.55	1.25	0.63	0.9	4.47	10.4	23.21
	Y (135°)	46.65	15.14	46.75	13.68	32.46	0.1	0.05	0.9	4.79	11.14	23.88
	T (90°)	48.38	14.61	47.98	13.78	30.21	0.41	0.2	0.9	4.62	10.75	22.22
Yes	I (180°)	46.84	16.32	48.31	13.76	34.84	1.48	0.74	0.9	5.16	12.0	25.63
	Y (135°)	47.13	14.93	47.53	13.15	31.69	0.41	0.2	0.9	4.72	10.99	23.31
	T (90°)	49.05	13.89	48.94	14.02	28.32	0.11	0.06	0.9	4.39	10.22	20.83

SD: Standard Deviation; CV (%): Coefficient of Variation; TE: Typical Error; CIM: Confidence Interval of the Mean; ICC: Intraclass Correlation Coefficient; SEM: Standard Error of Measurement; MDC90: Minimal Detectable Change at 90% Confidence; MDC%: Minimal Detectable Change Percentage.

**Table 4:** Differences between the preferred and non-preferred side.

Test	Position	Mean Preferred Side and Standard Deviation	Mean Non-Preferred Side and Standard Deviation	*p*-Value
Test ASH	I (180°)	78.88 (21.77)	77.86 (23.32)	0.633
		♂: 90.46 (23.46)	♂: 89.23 (25.37)	♂: 0.760
		♀: 66.14 (9.89)	♀: 65.35 (12.70)	♀: 0.818
	Y (135°)	64.48 (19.34)	64.39 (22.15)	1
		♂: 75.16 (21.26)	♂: 75.60 (24.06)	♂: 1
		♀: 52.74 (6.04)	♀: 52.05 (11.17)	♀: 0.625
	T (90°)	57.47 (14.00)	56.39 (15.02)	0.838
		♂: 65.86 (13.91)	♂: 64.95 (15.83)	♂: 0.895
		♀: 48.24 (6.30)	♀: 47.83 (6.21)	♀: 0.625
Test IASH	I (180°)	47.57 (16.05)	46.72 (15.89)	0.007
		♂: 55.86 (17.32)	♂: 54.65 (16.85)	♂: 0.054
		♀: 39.29 (7.12)	♀: 38.78 (6.89)	♀: 0.193
	Y (135°)	49.38 (13.45)	48.72 (14.02)	0.838
		♂: 56.92 (14.56)	♂: 56.11 (15.10)	♂: 0.705
		♀: 41.84 (8.32)	♀: 41.33 (8.05)	♀: 0.953
	T (90°)	44.21 (12.10)	43.67 (13.04)	0.562
		♂: 51.47 (13.08)	♂: 50.82 (13.89)	♂: 1
		♀: 36.95 (6.45)	♀: 36.52 (6.38)	♀: 0.625

**Table 5:** The strength ratios between the ASH and IASH tests.

Position	Mean Preferred Side and Standard Deviation	Mean Non-Preferred Side and Standard Deviation	*p* Value
I (180°)	Global: 1.732 (0.415)	Global: 1.791 (0.41)	0.229
	♂: 1.66 (0.32)	♂: 1.759 (0.424)	♂: 0.175
	♀: 1.81 (0.507)	♀: 1.826 (0.413)	♀: 0.770
Y (135°)	Global: 1.396 (0.239)	Global: 1.405 (0.271)	1.000
	♂: 1.415 (0.252)	♂: 1.369 (0.318)	♂: 0.831
	♀: 1.376 (0.236)	♀: 1.445 (0.219)	♀: 0.557
T (90°)	Global: 1.203 (0.241)	Global: 1.211 (0.199)	0.320
	♂: 1.176 (0.232)	♂: 1.196 (0.228)	♂: 0.240
	♀: 1.232 (0.26)	♀: 1.228 (0.173)	♀: 0.770

## Data Availability

The data that support the findings of this study are available on request from the corresponding author, P.H.-L.

## Abbreviations

The following abbreviations are used in this manuscript:

ASH	Athletic Shoulder Test
iASH	Inverse Athletic Shoulder Test
ICC	Intraclass Correlation Coefficient
SEM	Standard Error of Measurement
MDC90	Minimal Detectable Change at 90% Confidence
CV%	Coefficient of Variation Percentage
TE	Typical Error
NPF	Peak Force (Newton)
SD	Standard Deviation
CIM	Confidence Interval of the Mean
